# Rare metastasis of gastric cancer to the axillary lymph node: A case report

**DOI:** 10.3389/fonc.2022.995738

**Published:** 2022-10-25

**Authors:** Qingshun Zhu, Lei Li, Xuguang Jiao, Jinqiu Xiong, Shengyong Zhai, Guangxu Zhu, PeiPei Cheng, Jianjun Qu

**Affiliations:** ^1^ Department of Clinical Medical College, Weifang Medical University, Weifang, China; ^2^ Department of General Surgery, The First Affiliated Hospital of Weifang Medical University (Weifang people’s Hospital), Weifang, China

**Keywords:** gastric cancer, radical total gastrectomy, axillary lymph node metastasis, tumor markers, immunohistochemical staining

## Abstract

Lymph node metastasis of gastric cancer is more common, metastatic lymph nodes are often around the stomach, and metastasis is carried out in a certain order, but gastric cancer metastasis to axillary lymph nodes is very rare. Due to the small number of patients with this kind of metastasis, its clinical features and treatment are not very clear. We initially thought that the enlarged axillary lymph nodes were inflammatory lesions. Axillary lymph node biopsy was later diagnosed as gastric cancer metastases to axillary lymph nodes. The patient refused further treatment and died 11 months after the second operation because of multiple systemic metastases. We believe that metastasis of gastric cancer to axillary lymph nodes is rare and the prognosis is poor. In clinical work, the possibility of metastatic lymph nodes should be considered in patients with a history of gastric cancer with enlarged axillary lymph nodes.

## Introduction

Gastric cancer is a common malignant tumor of the digestive tract. Although morbidity and mortality have decreased in recent years, it is still the third largest cause of cancer death in the world (1). Advanced gastric cancer is often accompanied by lymph node metastasis and poor prognosis. Radical resection combined with standard lymph node dissection is still the main treatment for advanced gastric cancer (2). Lymph node metastasis of advanced gastric cancer often occurs in the lymph nodes around the stomach, and jump metastasis is rare, while axillary lymph node metastasis is rarer. According to the Japanese classification, axillary lymph node metastasis is considered to be distant metastasis of gastric cancer (3). Recently, we diagnosed and treated a patient with gastric cancer with left axillary lymph node metastasis one month after radical total gastrectomy. The report is as follows.

## Case presentation

**Figure 1:**
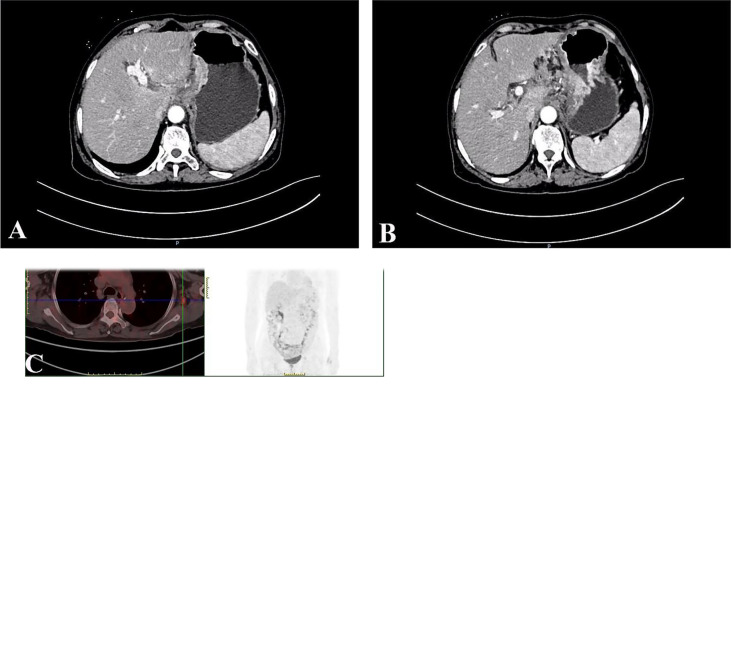
Abdominal enhancement CT showed obvious enhancement of the edge of the tumor in the arterial phase **(A)**; Abdominal enhanced CT showed enlarged retroperitoneal lymph nodes **(B)**; ^18^F-FDG PET-CT showed that the left axillary lymph node was enlarged, and the mass uptake of FDG increased slightly, but it was considered as an inflammatory lesion **(C)**.

**Figure 2:**
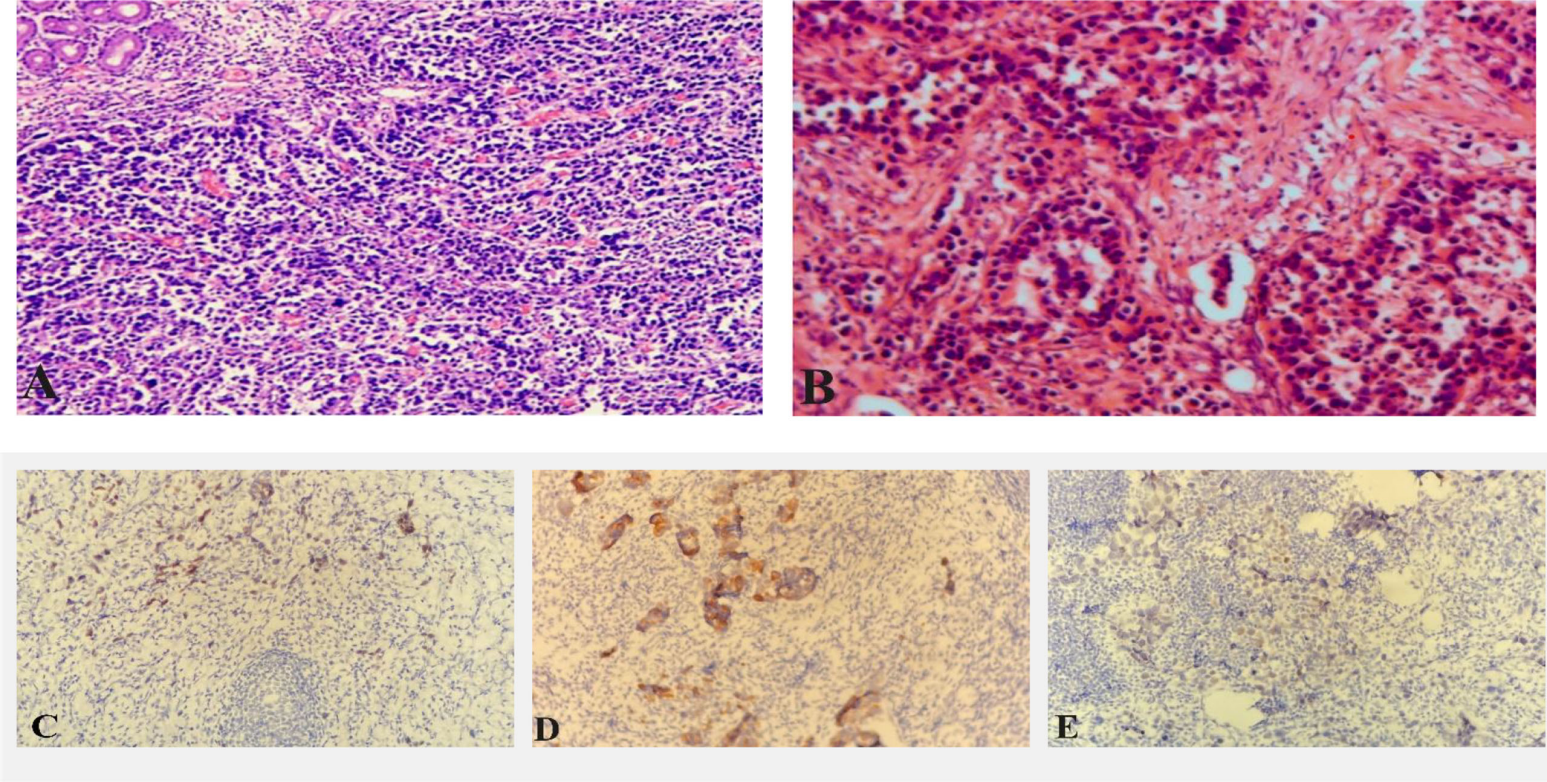
Pathological picture after radical total gastrectomy **(A)**; Pathological picture after radical axillary lymph nodes **(B)**; Immunohistochemical staining showed axillary lymph node tumor cells express CDX2 **(C)**; Immunohistochemical staining showed axillary lymph node tumor cells express CK20 **(D)**; Immunohistochemical staining showed some axillary lymph node tumor cells express SATB-2 **(E)**.

A 67-year-old female patient was admitted to the local hospital because of epigastric pain and discomfort for one month. She was diagnosed as a malignant tumor of the gastric body by electronic gastroscopy and biopsy pathology. The abdominal enhanced CT shows multiple lymph nodes enlargement in the abdominal and retroperitoneum in the outpatient clinic of our hospital (**Figures 1A, B** **)**. The patient received six cycles of chemotherapy in another hospital (SOX regimen for 1 cycle; S-1 and oxaliplatin. Paclitaxel, oxaliplatin, S-1, and Sintilimab for 5 cycles). After the end of chemotherapy, the effect of chmotherapy was evaluated as partial remission (PR). The patient asked for surgical treatment in our hospital. Physical examination showed that the abdomen was flat, the abdominal muscles were soft, the upper abdomen was mild deep tenderness, there was no rebound pain, and there was no obvious abdominal mass. Laboratory results showed that hemoglobin content decreased: 97g/L (normal range 110-150g/L). CA72-4:4.90U/mL (normal range 0-6.9U/mL), AFP: 1.30ng/mL (normal range 0-8.1ng/mL), CEA:1.13ng/mL (normal range 0-10ng/mL), CA199:17.71U/mL (normal range 0-37U/mL), CA125:2.85U/mL (normal range 0-30.2U/mL). There was no significant increase in serum tumor markers. After the end of neoadjuvant chemotherapy, we performed a PET-CT examination for the patient. ^18^F-FDG PET-CT showed that the mass showed changes after chemotherapy, slight thickening of the lesser curvature of the stomach, the mass did not significantly absorb FDG, and it was found that the left axillary lymph node was enlarged, and the mass uptake of FDG increased slightly, but it was considered as an inflammatory lesion (**Figures 1C**). After multidisciplinary tumor consultation, we decided to perform the radical total gastrectomy on the patient, and regular examination of the enlarged lymph nodes in the left axilla. After obtaining the consent of the patient and her family, the patient underwent radical total gastrectomy (Roux-en-Y digestive tract reconstruction) in August 2021. Postoperative pathology showed that the area of ulcerative gastric cancer was about 4 × 3cm. The main tumor cells were poorly differentiated adenocarcinoma, local invasion of the deep muscular layer of the gastric wall, and tumor cells can be seen in the lymphatic vessels but no definite nerve invasion. Lauren’s classification was the diffuse type (**Figure 2A**). Only one of the 21 lymph nodes had metastasis, which was located on the lesser curvature of the gastric wall, and no obvious tumor metastasis was found in the rest of the lymph nodes (ypT2N1M0 IIA). Immunohistochemical staining showed that tumor cells expressed CK8/18, individual cells expressed Syn, and did not express CgA, CD56, SALL4, Oct3/4, C-erb-B-2, and Ki-67 proliferative index was approximately 90%. The patient recovered smoothly without obvious postoperative complications and was discharged 13 days after radical total gastrectomy.

**Figure 3:**
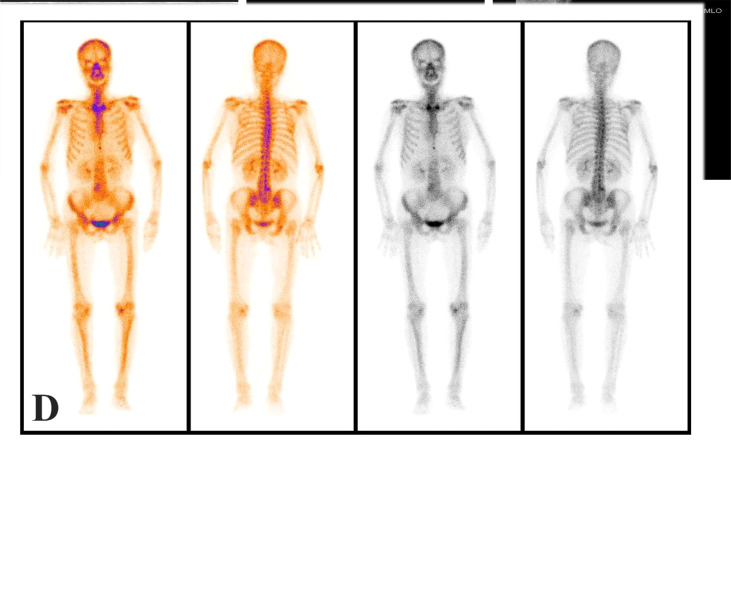
Axillary ultrasound showed enlarged axillary lymph nodes **(A)**; The mammary gland molybdenum target X-ray was not found obvious breast mass **(B, C)**; No obvious mass was found in whole-body bone scintigraphy **(D)**.

One month after radical total gastrectomy, the patient found that the left axillary mass grew faster than before and was accompanied by the limitation of left upper limb movement. Physical examination showed that the left axillary mass was about 4 × 2cm in size, hard, had an unclear boundary, and had a poor range of motion. Ultrasound examination of the bilateral breast and axilla showed that there was no obvious mass in the bilateral breast, and several hypoechoic lesions were found in the left axilla, the size of which was about 4.3 × 1.9cm, the boundary was clear, the cortex was thickened, the medulla was eccentric and the blood flow signal was abundant (**Figure 3A**). No obvious abnormality was found in mammary gland molybdenum target X-ray (**Figures 3B, C** **)**, mediastinal and supraclavicular enlarged lymph nodes were not found in CT, and no obvious bone metastasis in whole-body bone scintigraphy (**Figure 3D**). The patient underwent the axillary lymph node biopsy in October 2021. During the operation, the enlarged lymph nodes were located next to the axillary vein, fused into clumps, hard texture, and closely combined with the surrounding tissues. Intraoperative frozen sections showed that there were 5 lymph nodes in the left axilla, and all of them had cancer metastasis. After that, we performed radical axillary lymph node dissection and 14 of the 18 lymph nodes had metastases. Pathology showed that the tumor cells were poorly differentiated adenocarcinoma. immunohistochemical staining showed that most of the tumor cells expressed Caudal-type homeobox 2 (CDX2), CK20, GATA binding protein 3 (GATA-3), and a small amount of sequence-binding protein (SATB) 2 and Mucin-5AC (MUC5AC), but no expression of CK7 and TTF-1 was found (**Figures 2B–E**). After communicating with pathologists, considering the immunohistochemical results and the history of gastric cancer, we considered that the left axillary lymph node tumor was metastasized by gastric cancer.

### Outcome and follow-up

**Figure 4:**
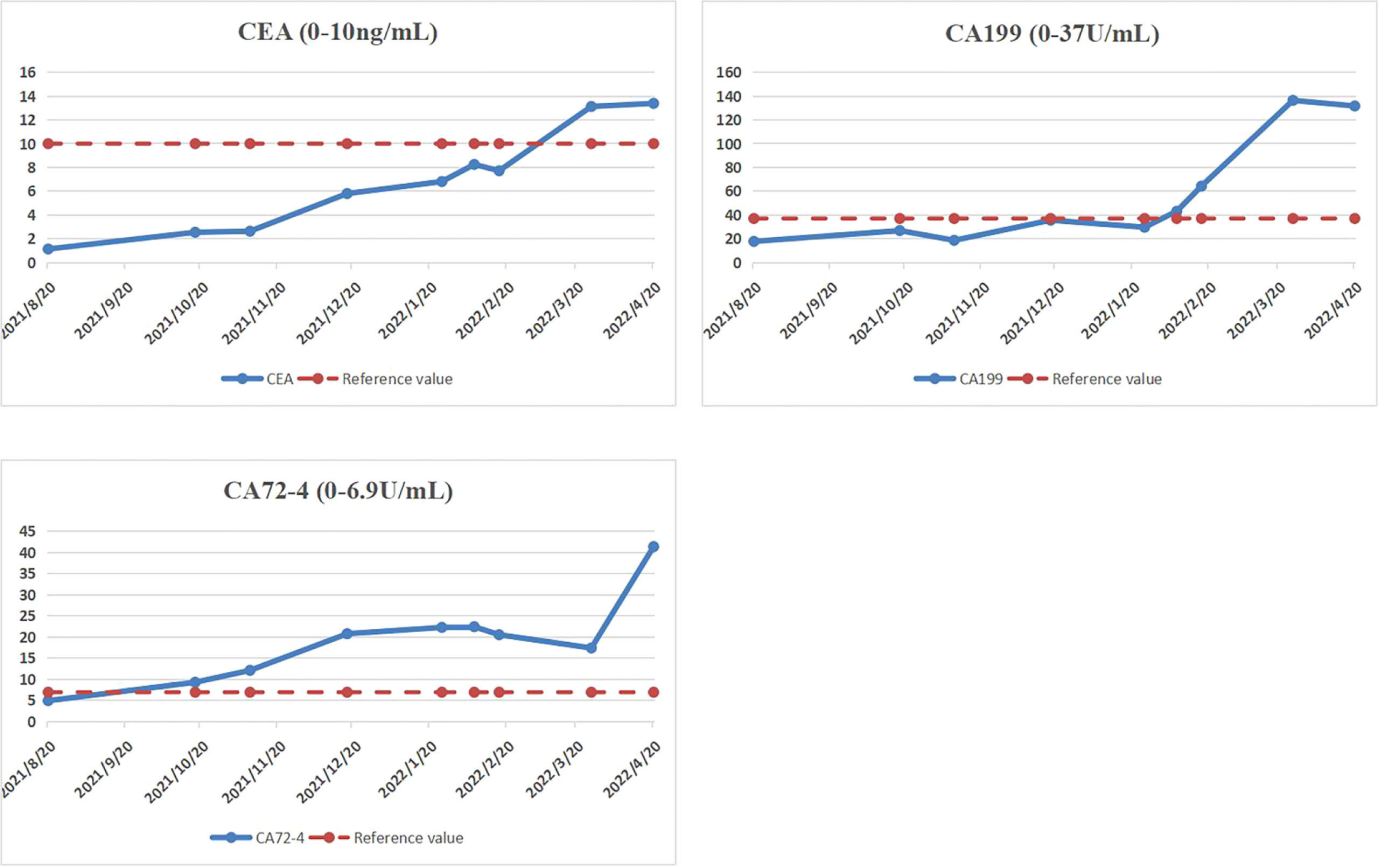
CEA, CA19-9, and CA72-4 increased gradually after radical axillary lymphadenectomy.

The patient received docetaxel and fluorouracil chemotherapy after radical axillary lymph node dissection, and a progressive increase in CEA, CA19-9, and CA72-4 was found **(** **Figure 4** **)**. Three months after the second operation, the MR examination of cervical and thoracic vertebrae due to back pain revealed secondary malignant tumors of the spine, but the patient refused to undergo whole-body bone imaging. CT of the chest and abdomen showed double clavicular and mediastinal enlarged lymph nodes, considering the malignant tumor. But the patient refused any treatment and died 11 months after the second operation.

## Discussion

At present, although most patients with gastric cancer have received radical surgery and comprehensive treatment such as neoadjuvant chemotherapy, conversion therapy, or adjuvant chemotherapy, but some patients still have recurrence and metastasis of liver, lung, bone, or lymph nodes during postoperative follow-up (4). In patients with gastric cancer, axillary lymph node metastasis is very rare. Through literature search, we found 3 reports of axillary lymph node metastasis in gastric cancer (two of which do not have English abstracts). Kobayashi reported a case of left axillary solitary metastasis 21 months after radical distal gastrectomy and radical axillary lymph node dissection, and no tumor recurrence was found one year after the operation (5). In addition, cases of gastric malignant tumors due to the discovery of enlarged axillary lymph nodes were also reported, but the details were not reported in detail.

Postoperative axillary lymph node metastasis of gastric cancer should be differentiated from occult breast cancer (OBC). OBC refers to breast cancer with no primary breast lesion, but with axillary lymph node metastasis or other distant metastasis as the first symptom (6). Histopathological examination of axillary enlarged lymph nodes can make a definite diagnosis. Histopathological examination can not only reveal the pathological type of metastatic lesions but also further reveal the source of primary lesions by immunohistochemical staining (7). In this case, tumor cells expressed specific markers of digestive tract tumors (CDX2, CK20). Combined with the history of gastric malignant tumor, we considered that the left axillary lymph node tumor was metastasized by gastric cancer. Malignant tumors may increase tumor markers in the blood or body tissue. In addition, the increase of many specific tumor markers may indicate tumor recurrence and metastasis. Most tumor markers are effective prognostic tools that can be used to identify the risk of recurrence or metastasis (8). CA72-4 is a mucin-like glycoprotein that exists on the surface of many cancer cells. CA72-4 detection has good specificity for gastric cancer and can be used to determine the recurrence of GC and follow-up after treatment (9). CEA is the most widely used and most frequently used marker in digestive system tumors, the level of CEA is increased in some patients with advanced gastric cancer (10). Previous studies have found that the positive rate of CEA is 21.1%, the positive rate of CA19-9 is 27.8%, and the positive rate of CA72-4 is 30.0%. These three markers were significantly correlated with tumor stage and patient survival. Serum markers are not helpful for early cancer, but they are helpful in detecting recurrence and distant metastasis, predicting patient survival, and postoperative monitoring (8). In addition, the increased expression of Ki-67 was associated with the proportion of metastatic lymph nodes in the total number of lymph nodes and the advanced stage of the tumor (11). In this patient, before radical axillary lymph node dissection, the serological tumor markers were in the normal range and considering that it is very rare for gastric cancer to metastasize to axillary lymph nodes, we misjudged the patient’s condition. It also suggests that we should consider the possibility of metastasis in patients with gastric cancer with enlarged axillary lymph nodes and biopsy of enlarged lymph nodes is a method to determine the primary tumor.

The lymph node metastasis of gastric cancer mainly occurs gradually along the lymph node drainage pathway, but the gastric malignant tumor shows cross-regional distant lymph node jump metastasis, which is difficult to explain by the conventional lymph node pathway. Kobayashi considered that the primary tumor may have invaded the lymphatic vessels of the chest wall because the lymphatic drainage of the axillary lymph nodes comes from the subcutaneous or intercostal lymphatic vessels of the chest wall (5). Parungo detected that the celiac lymph nodes can be drained directly to the chest wall lymph nodes by using a fluorescent tracer and then to the thoracic lymph nodes (12). We speculate that the axillary lymph node metastasis of gastric cancer may occur in the following ways: the first may be that the tumor cells invade the thoracic duct, then invade the blood circulation, and enter the left subclavian lymphatic vessel through the left subclavian vein. And to the axillary lymph node drainage direction countercurrent, resulting in axillary lymph node metastasis. The second possibility is that the tumor cells invade the lymphatic vessels of the abdominal wall or chest wall, resulting in axillary lymph node metastasis, because the superficial lymphatic vessels of the sub umbilical abdominal wall flow downward into the inguinal lymph nodes, and the supraumbilical lymphatic vessels flow upward into the axillary lymph nodes (13). The third possibility is that the tumor cells directly invade the blood circulation and when passing through the axilla, they are captured by the axillary lymph nodes and reproduce and grow in the axillary lymph nodes, resulting in axillary lymph node metastasis. In this case, we speculated that the tumor invaded the lymphatic vessels of the chest wall and then metastasized to the left axillary lymph nodes through the thoracic duct.

Lymph node metastasis of gastric cancer is an important index affecting the prognosis of gastric cancer. With the increase in the number of metastatic lymph nodes, the overall survival rate of patients with gastric cancer decreased significantly (14). At the same time, the recurrence rate of patients with positive lymph nodes was significantly higher than that of patients with negative lymph nodes (15). Axillary lymph node metastasis of gastric cancer is considered to be a kind of distant metastasis, which is related to poor prognosis. Based on the relevant literature, it is not clear which treatment is more helpful to improve the prognosis of patients after surgical resection, adjuvant chemotherapy, or direct systemic chemotherapy. Pavlidis considered that metastatic adenocarcinoma, including solitary axillary lymph nodes, can be treated by surgery (16). Zhao reported that adequate lymph node dissection is essential for skip lymph node metastasis (17). Kobayashi reported that the left axillary lymph node metastasis occurred after the gastric cancer operation, there was no definite recurrence or metastasis within one year after radical lymphadenectomy (5). Nashimoto reported a case of gastric cancer who survived for more than 6 years after abdominal para-aortic lymph node dissection (18). Chieco reported a case of solitary metastasis of left axillary lymph nodes after operation of right colon cancer and there was no recurrence within 1 year after radical resection (19). Therefore, we speculate that postoperative axillary lymph node metastasis of gastric cancer can be considered as a local manifestation of systemic metastasis, and surgical resection of local recurrent lesions is a more effective treatment at present. However, unlike in other cases, axillary lymph node metastasis occurred one month after operation in this patient, so we speculate that the tumor cells have a high malignant potential. Therefore, we give adjuvant chemotherapy after the radical axillary lymphadenectomy.

Axillary lymph node metastasis is one of the sites of distant metastasis of gastric cancer. In the preoperative evaluation and postoperative reexamination of patients with gastric cancer, attention should be paid to axillary lymph node examination. The biopsy of enlarged lymph nodes is a good method to determine the primary tumor. For this patient, we underwent radical resection of metastatic lymph nodes, but the treatment of gastric cancer with axillary lymph node metastasis is still controversial. Further cases may be needed in the future to determine the appropriate surgical intervention and the duration of chemotherapy to determine which treatment is more beneficial to improve the prognosis of patients with postoperative axillary lymph node metastasis of gastric cancer.

## Conclusion

Gastric cancer with axillary lymph node metastasis is very rare. Pathological examination of enlarged lymph nodes is a good method to identify primary tumors, which can improve the accuracy of diagnosis and avoid excessive treatment. At present, surgical resection of enlarged lymph nodes is a more effective treatment, but more studies are still needed to determine which treatment is more beneficial to improve the prognosis of patients with gastric cancer with axillary lymph node metastasis.

## Data availability statement

The original contributions presented in the study are included in the article/supplementary material. Further inquiries can be directed to the corresponding author.

## Ethics statement

The studies involving human participants were reviewed and approved by the Weifang People’s Hospital Ethics Committee. The participants provided their written informed consent to participate in this study. Written informed consent was obtained from the individual(s) for the publication of any potentially identifiable images or data included in this article.

## Author contributions

JQ: guarantees the integrity of the entire study and edited the manuscript. QZ and LL: prepared and edited the manuscript. XJ, JX, SZ, GZ and PC: performed the literature research, data analysis, and text proofreading. All authors contributed to the article and approved the submitted version.

## Funding

This study is supported by the Medical and Health Science Technology Development Program in Shandong Province (202104080159) and Science and Technology Development Program in Weifang City (2019YX002 and 2021YX007) and Scientific Research Project of Weifang Health Commission (WFWSJK-2021-028).

## Acknowledgments

I would like to express my gratitude to all those who helped me during the writing of this manuscript and their efforts in the management of this patient.

## Conflict of interest

The authors declare that the research was conducted in the absence of any commercial or financial relationships that could be construed as a potential conflict of interest.

## Publisher’s note

All claims expressed in this article are solely those of the authors and do not necessarily represent those of their affiliated organizations, or those of the publisher, the editors and the reviewers. Any product that may be evaluated in this article, or claim that may be made by its manufacturer, is not guaranteed or endorsed by the publisher.
